# Hounsfield units and fractal dimension (test HUFRA) for determining PET positive/negative lymph nodes in pediatric Hodgkin’s lymphoma patients

**DOI:** 10.1371/journal.pone.0229859

**Published:** 2020-03-19

**Authors:** Radosław Chaber, Mateusz Łasecki, Karol Kuczyński, Rafał Cebryk, Justyna Kwaśnicka, Cyprian Olchowy, Kornelia Łach, Zbigniew Pogodajny, Olga Koptiuk, Anna Olchowy, Paweł Popecki, Urszula Zaleska–Dorobisz

**Affiliations:** 1 Clinic of Pediatric Oncology and Hematology; Medical Faculty, University of Rzeszow, Rzeszow, Poland; 2 Medical University of Wroclaw, Wroclaw, Poland; 3 The State School of Higher Education in Chełm, Chełm, Poland; 4 Institute of Computer Science, Maria Curie-Sklodowska University, Lublin, Poland; 5 Department of Pediatric Bone Marrow Transplantation, Oncology and Hematology, Wroclaw Medical University, Wroclaw, Poland; 6 Department of Oral Surgery, Wrocław Medical University, Wrocław, Poland; 7 Affidea Center of Positron Emission Tomography and Computed Tomography, Wrocław, Poland; 8 Radiology Department, Lower Silesian Oncology Center in Wrocław, Wrocław, Poland; 9 Department of Experimental Dentistry, Wroclaw Medical University, Wroclaw, Poland; 10 Departament of Dental Surgery, Wroclaw Medical University, Wroclaw, Poland; 11 Department of Radiology, Medical University of Wroclaw, Wroclaw, Poland; Karmanos Cancer Institute, UNITED STATES

## Abstract

**Objectives:**

We had developed a method that can help detect and identify lymph nodes affected by the neoplastic process. Our group evaluated the fractal dimension (FD) and X-ray attenuation (XRA) of lymph nodes in HL and compared to their metabolic activity as measured by 18F-FDG-PET examination.

**Methods:**

The training set included 72 lymph nodes from 31 consecutive patients, and the tested set of 71 lymph nodes from next 19 patients. The measurement of FD of each lymph node was performed before the start of therapy using original software. X-ray attenuation (XRA) expressed in HU (Hounsfield Units) from CT scans was compared with the metabolic activity of the lymphatic nodes, measured by 18F-FDG-PET examination.

**Results:**

Significant differences were observed between XRA_max_ and FD_max_ values in assessing the PET(+) and PET(-) nodes. All nodes were scored from 0 to 2. The HUFRA test properly qualified 95% with a score of 2 and 0 points as PET(+) or PET(-).

**Conclusion:**

The HUFRA test can differentiate about 70–80% of lymph nodes as PET(+) or PET(-) based solely on the CT examination. It can be useful in patients who were not subjected to 18FFDG-PET/CT examination before the treatment, or who had an unreliable result of 18F-FDG-PET/CT with further research requirements.

## Introduction

Contemporary treatment of Hodgkin's lymphoma (HL) in children and adolescents is based on chemotherapy (CTx), and in some cases, on CTx combined with radiotherapy (RTx). A significant reduction of treatment is currently considered in cases without manifesting risk factors due to good results of HL treatment [1–3]. Adapted risk therapy strategy allows reducing toxicity, which is a major factor, contributing to a considerable decrease in the long-term quality of life and life expectancy in patients treated for HL in childhood [4]. It is noted that the majority of late complications in pediatric population are caused by radiotherapy [5–7].

After histopathological confirmation of HL, the therapeutic scheme is dependent on the FDG-PET/CT result [8], a procedure to visualize the metabolic activity of tissues. In clinical cases of patients after chemotherapy (2 cycles) in whom no satisfactory regression / clinical response of the disease was found, radiotherapy focused to the involved organs (almost always lymph nodes) will be necessary. For this reason, we believe that there is a need for accurate diagnosis and identification of malignant lymph node areas. Due to a large number of occupied lymph nodes, histopathological evaluation of each potentially malignant lymph node would be highly troublesome for physicians, parents and the child. PET-CT is a study with similar predictive abilities but much less problematic than histopathology. Typical parameters obtained from PET-CT are those used in the Deauville scale [9]: lymph node involvement, maximum diameter, and 18FDG uptake in tissues.

"Classical" CT reports usually describe lymph nodes using only the following parameters: position, size, shape, while information about tissue density (Hounsfield units) is usually omitted. However, a detailed analysis of CT images allows for the determination of additional tissue parameters, such as homogeneity/heterogeneity, which can also serve as a source of useful information [10]. In one of our previous studies, we demonstrated the practical usefulness of HU values in HL cases in the pediatric population [11]. We had shown that density values (measured in Hounsfield units) are much higher in malignant lymph nodes compared to "benign" ones when the standard metabolic criteria (Deauville scale) are considered as the reference point. This difference was observed both in the primary study and after two cycles of chemotherapy.

The term "fractal" is a measure of self-similarity that describes the persisting "similarity" of an object's images despite the change in zoom scale. It is also infinitely subtle because it shows subtle details even at high magnifications. The self-similar dimension, namely fractal dimension (FD), is defined as a logarithm to the base of a similarity scale equal to the number determining how many times the initial figure is bigger than a similar figure. Many natural objects (mountains, coasts, trees, clouds, butterflies’ wings, eye iris, metal corrosion, etc.) are self-similar [12–14]. That is why fractal analysis is a reasonable choice where natural objects are concerned. Assessing the FD in the CT images of some neoplasms maximizes the information obtained from current standard images and can be implemented into the clinical workflow [15,16]. In our previous publication, we described the algorithm of the software which allows measuring of the FD in CT scans of affected lymph nodes [17]. Shortly, in the presented solution, a semi-automatic procedure was implemented. A popular ITK*a* ContourExtractor2DImageFilter procedure is employed. It uses the “marching squares method”, which is a special case of the well-known marching cubes algorithm. The iso-valued contours (zero or more) are found of the input 2D image (either binarized or grayscale), for a given intensity. The contours are closed paths unless they intersect with the image edge. The fractal dimension is then calculated for a square area surrounding the previously detected contour or selected manually by a user [17].

In the current research, we attempted to assess the value of FD of a lymph node and its X-ray attenuation (XRA) when measure with HU in CT scans in HL because the auxiliary parameters of the lymphatic nodes allow assessment of their metabolic activity as it is measured by 18F-FDG-PET/CT examination.

## Material and methods

### Patients

To the study, 51 consecutive patients with a diagnosis of HL were enrolled from June 2009 to June 2015. The participant’s characteristics are presented in Table 1. All histological diagnoses were confirmed by a referenced histopathologist who is experienced in diagnosing HL. A retrospective analysis encompassed 143 supradiaphragmatic lymph nodes, which were assessed by 18F-FDG-PET/CT and confirmed the diagnosis. All analyzed 18F-FDG-PET/CT examinations were routinely carried out in the diagnostic process for HL. For the purpose of the present study, they were retrospectively evaluated. Patient data were re-identified ensuring HL condition and diagnosis. Informed consent was obtained from all patients or their guardians before the treatment. This retrospective study was conducted in compliance with international regulations for the protection of human research subjects and was approved by the regional scientific ethics committee (KB-433/2013 Wroclaw Medical University).

**Table 1:** Clinical characteristics of patients.

	training set	tested set
Number of patients	31	19
Boys/girls	14/17	10/9
Age, range (median)	8–18 (15)	5–17 (14.5)
HL type		
NS	28	19
MC	1	0
LD	2	0
stage I	1	0
stage II	13	15
stage III	7	2
stage IV	10	2
Number of LN analyzed	72	71
PET(+)/PET(-)	47/25	31/40

HL, Hodgkin lymphoma; LN, lymph node

The patients participating in the research study were divided into 2 groups. Overall, 31 consecutive patients were randomly qualified to training set and next 19 to the test set. The total number of supradiaphragmatic lymph nodes assessed in each group, according to the following principles, was 72 and 71, respectively. In the lymph nodes considered, the maximal values of the parameters such as a maximum standardized uptake value (SUV_max_), maximum fractal dimension (FD_max_), and maximum X-ray attenuation (XRA_max_) were determined. The XRA_max_ was defined as a ratio of XRA_max_ of a lymph node and XRA_max_ of erector spinae muscles and was expressed in HU.

### Analysis of CT images

Unenhanced 18F-FDG-PET/CT examinations were performed in the reference group using a 16-row detector GE Discovery STE16 scanner (GE Healthcare, Milwaukee, United States) with variable voltage (100–140 keV) and X-ray tube current (10–150 mAs), single collimation width (SCW) 0.625–1.25, total collimation width (TCW) 10.0–20.0, and spiral pitch factor (SPF) 1.125–0.75. Examinations in the test set were carried out with PET-CT GE Discovery IQ with 512-row detector, voltage 80–120keV, X-ray tube current (31–208), SCW 1.25, TCW 20, SPF 1.375. All scans were performed before the start of therapy.

Axial sections were used for the assessment. The preference was given to those having the thinnest layers which ranged from 1.25 to 3.75 mm, depending on the study/patient. This allowed for a reduction of the occurrence of the artifact, such as partial volume artifact and partial volume averaging artifact [18], which could be a source of errors during qualification of a lymph node as healthy or affected and may influence the measurement of FD, particularly in small structures such as lymph nodes. For the same reason, the FD assessment based on lymph node counting was abandoned. The research was focused solely on the FD assessment of the lymph node structure, based on the reconstruction of numerical data, including the XRA degree of a given tissue and displayed as a series of images with different grayscale build from a set of pixels.

All measurements were performed using axial images–a component of the CT scan of 18F-FDG-PET/CT examination. Lymph nodes were well visible on the multiplanar reconstructions (MPR) in other planes, but those with poor differentiation on axial images were excluded from the analysis.

**Fig 1:**
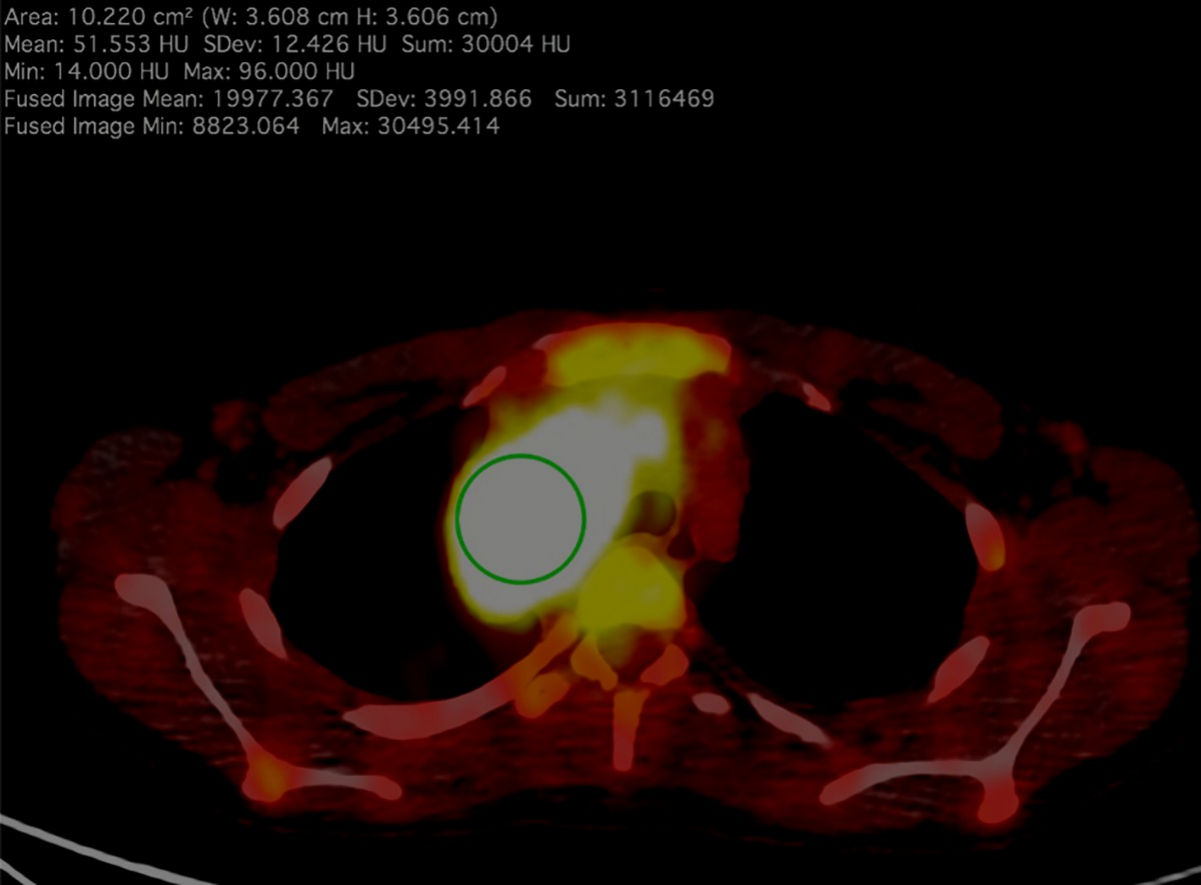
Fused PET-CT image of a female adolescent with HL; the measurement shows the highest value of both SUV_max_ (30.5) and XRA_max_ (96.0). The arithmetic mean of XRA_max_ was a subject to the statistical analysis.

**Fig 2:**
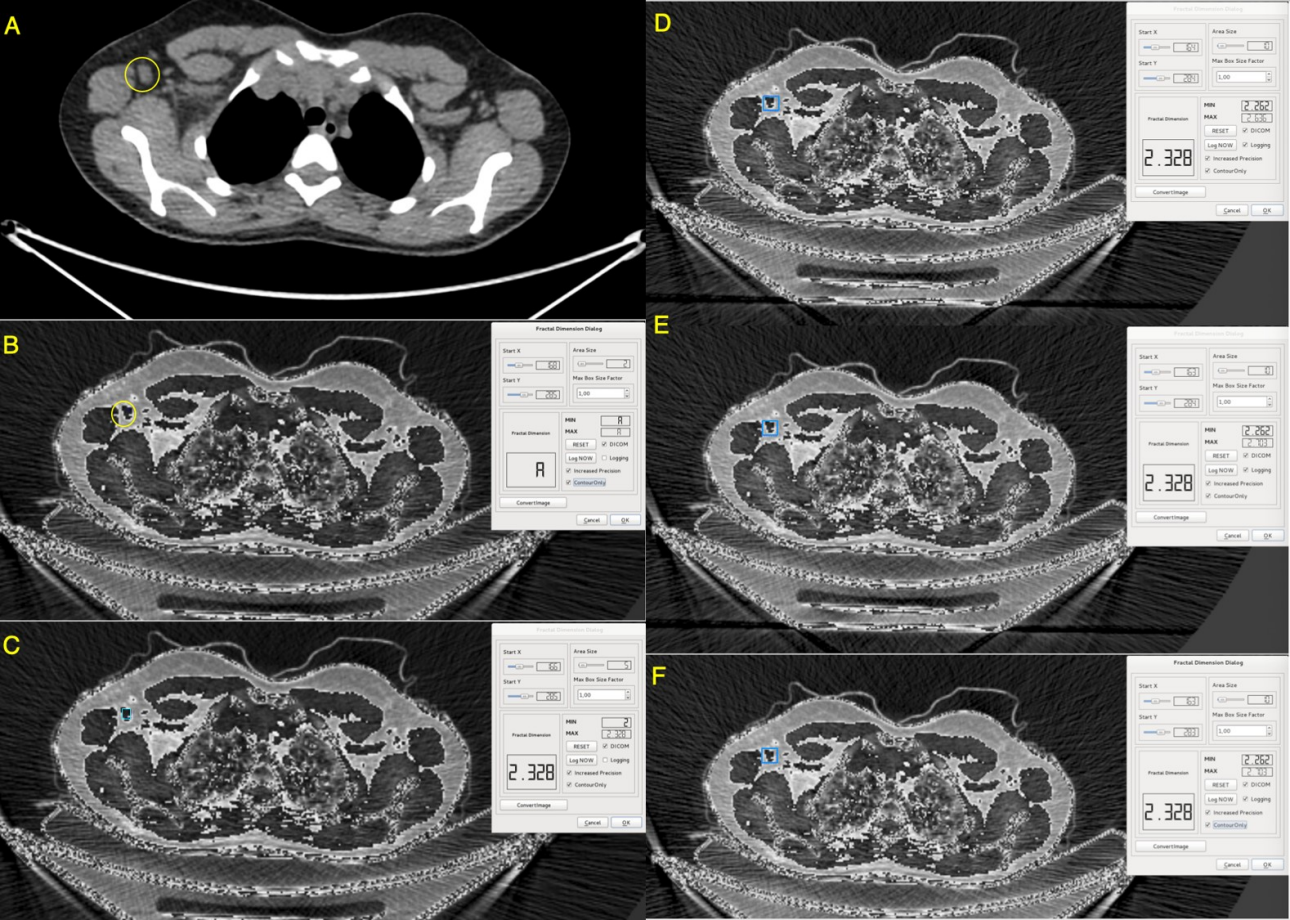
A. Image of the DICOM file in a standard workstation (in this case OSIRIX MD), a single enlarged axillary lymph node is marked in yellow (group 1 according to Berg's classification). B. The image of the same DICOM file in our workstation assessing the fractal dimension, the image has less smooth contours than the original one; the yellow area is marked with the same area as the image "A". On the right side, zero fractal measurements from previous measurements (min, max, Fractal Dimension). C. The blue frame shows a sample from an image size 5 (area size), covering only the lymph node area, which, however, due to too small "area size" was not further evaluated. D, E, F. The blue frame shows a sample from an area size image, covering the lymph node area and adjacent connective tissue, from three consecutive measurements. The average fractal dimension in all of them remained constant (2,348), while the max value (2.626–2.703) differs as a result of a slight shift of the frame position between measurements (human error).

The referenced scans of the CT component of 18F-FDG-PET/CT in the axial plane were selected based on the visual evaluation of the fused images. The examination of the sections with the greatest brightness was performed with a ROI tool to obtain the highest SUV_max_ (Fig 1). When the SUV_max_ of a lymph node/tumor was equal to those of the adjacent normal tissues, the long axis image of the structure with the greatest dimension was deemed the basic cross-section. The scan, including SUV_max_ (or the greatest length in the axial plane), became a starting point for further measurements (Fig 2). For the assessment of the XRA_max_ value in bigger structures, apart from the basic cross-section, 2 neighboring images in the cephalad and 2 in the caudal direction (5 sections in total) were assessed. The increase in the number of measurements resulted from the possibility of a small image shift in PET compared to the CT images [19]. Using the above-described method for the assessment of the small lymph nodes, outer cross-sections often did not include any part of the selected node. In such cases, the analysis had combined the basic cross-section and at least one neighboring image, cephalad or caudal (Fig 2).

### Principles for the evaluation of lymph nodes

Due to the considerably more frequent involvement of supradiaphragmatic lymph nodes in children, thoracic and neck lymph nodes were included in the present analysis.

Lymph nodes were evaluated according to the Society for Pediatric Oncology and Hematology’s GPOH-HD 2002 study and Lugano criteria [9,20,21] independently by 2 radiologists and a nuclear medicine specialist. The Surveillance, Epidemiology, and End Results program (SEER) database guidelines with minor modifications were adopted for the anatomic division of lymph nodes [22]. The preferred normal lymph node locations were: axillary (level 1 according to Berg), cervical (level 5 according to Som et al.), or the mandibular angle [23,24]. Lymph nodes with the SUV value of 4 or 5 points on the Deauville scale were included in the group of PET-positive (PET+) lymph nodes. In our research, this assumption allowed inclusion of the lymph nodes with SUV_max_ of ≥10 (the conclusion was based on the data from the literature [25,26] and the fact that the highest metabolic activity of the liver was equal to SUV_max_ of 9.8 in the corresponding patients). The lymph nodes with SUV_max_ between 1 and 3 (Reference-Deauville scale) were classified as PET negative (PET-).

The XRA measurement was made on an axial section of the node with the thickest cortical layer. The fatty hilum was excluded from the measurement as it would decrease the average XRA value.

The XRA values were assessed in all cases. Specifically in the sections where the fused image showed the highest SUV_max_ value and additionally, in the 2 adjacent sections (due to the possibility of a small image shift in PET compared to the CT images) [19,27]. For all analyzed lymph nodes, the limit of the greatest XRA value was selected which did not exceed 200 HU because this value indicates the presence of calcification [28]. In order to eliminate non-specific inter-individual variability related to different water and body fat content, the XRA lymph node/XRA muscle (LN/M) ratio was introduced. This value is obtained by dividing the maximum lymph node HU value by the HU value of the right erector spinae muscles. The obtained results are also independent of the keV and mAs parameters used in the study. The measurements of the right erector spinae muscle were performed in axial sections of the CT at the arising area of celiac and superior mesenteric arteries. The detailed description of a ROI selection and determination of XRA_max_ of the right erector spinae muscle was provided in our previous publication [11].

The measurement of FD of a lymph node was carried out using original software, which was previously described [17]. The measurement of FD value was based on the modified principles provided by Raja et al. [10]:

- The rectangular shape of ROI was used,
- The measurement of FD was performed manually by a radiologist experienced in evaluating and describing lymphatic nodes lesions in children,
- The ROI excluded lungs, air spaces in the gastrointestinal and respiratory tract, bones and any foreign bodies (e.g., metallic surgical clips),
- The size of ROI for normal lymph nodes of the largest dimension (<1.0 cm) was optimally 10x10 pixels, excluding any other tissues than nodular or fatty in the surveyed measuring area. In the case of the occurrence of other tissues, the ROI was reduced to the borderline value of 6x6 pixels. The lymph nodes of <1.0 cm were measured along with the adjacent connective tissue and a fatty hilum was typical for normal nodes. The measurement of FD including adjacent fatty tissue was required due to the fact that FD of the tissue around the tumor is changing due to the metabolic activity of cancer [29].
- In the case of big lymph nodes (the largest dimension of >2.0), the size of ROI was relying on the size on the node measured, with the goal to examine the largest area possible, not smaller than 12x 12 pixels. In this group of nodes, ROI used for the assessment of FD, had always covered as the largest area as possible within the assessed lesion. This area had no contact with its contour. The fatty hilum, if present, was excluded during the measurement.

In the case of small structures/organs, poor repeatability of numerical values may take place [30], which was confirmed by the present research. In order to minimize the risk of obtaining a false-positive result of the FD measurement or an outlier, in the large structures, 3 independent measurements of FD in each of the 5 surveyed regions were carried out. The median of those measurements was providing the ultimate value. In the case of normal lymph nodes, if the number of FD values was even, an additional measurement of FD in the section of the highest value of SUV and/or the greatest dimension in the long axis was carried out.

### Statistical analysis

The distributions of XRA_max_ LN/M and FD_max_ values were other than normal, for this reason, they were compared within PET(+) and PET(-) groups using the Mann–Whitney U test. The optimal cut-off point for distinguishing normal nodes from HL-affected nodes was determined using receiver operating characteristic analysis (ROC) by implementing the Youden index. The level of significance was p <0.05. The calculations were performed using Dell Inc. (2016). Dell Statistica (data analysis software system), version 13. software.dell.com.

## Results

**Fig 3:**
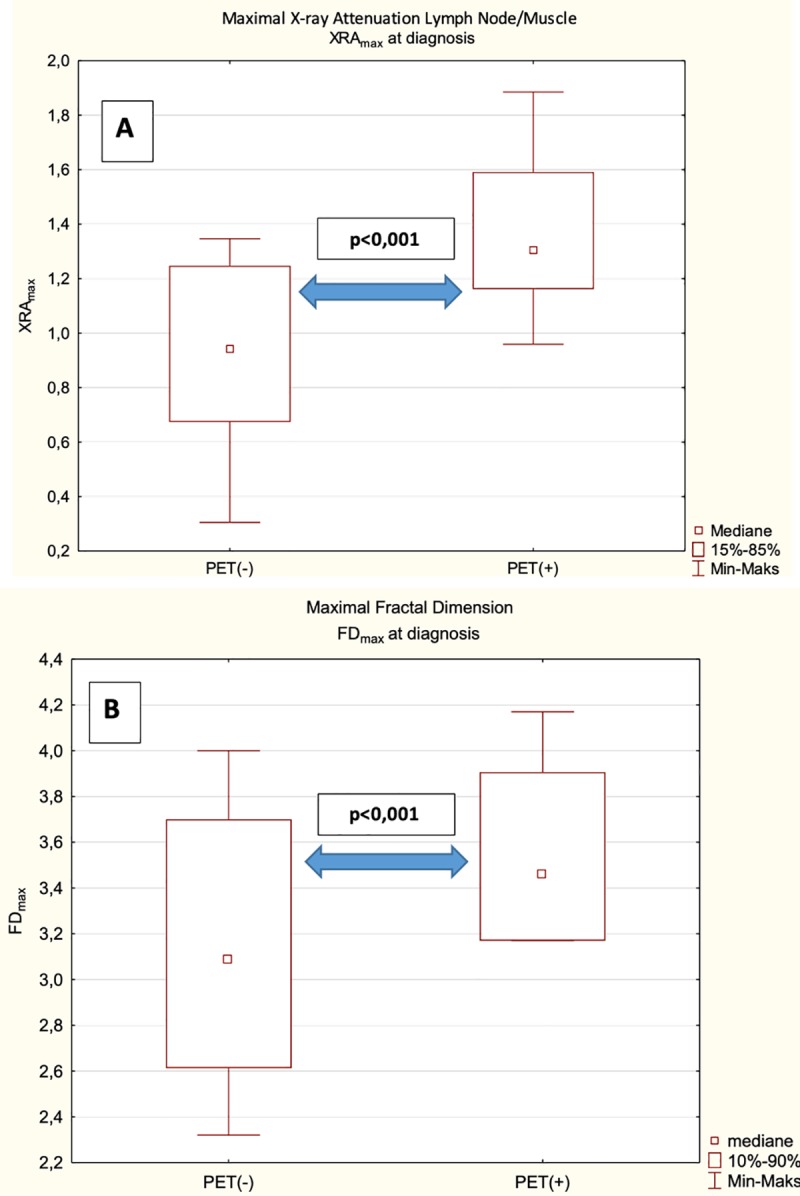
Maximal X-ray attenuation lymph node/muscle (A) and fractal dimension (B) at diagnosis in the training set.

The values of XRA_max_ LN/M, FD_max_ and SUV_max_ determined during 18F-FDG-PET/CT examination at diagnosis in the training set are shown in Table 2 and Fig 3.

**Table 2:** XRA_max_ LN/M, FD_max_ and SUV_max_ as determined with 18F-FDG-PET/CT at diagnosis in the training set.

	SUV_max_			XRA_max_ LN/M			FD_max_	
	median	range	SD	median	range	SD	median	range
**PET(+)** (n = 47)	21.00	10.4–46.60	8.37	1.30	0.96–1.88	0.24	3.46	3.17–4.17
**PET(-)** (n = 25)	2.10	1.00–9.60	2.52	0.94	0.30–1.35	0.27	3.09	2.32–4.00

LN, lymph node; SUV_max_, maximum standardized uptake valve; FD_max_, maximum fractal dimension; XRA_max_, maximum X-ray attenuation.

**Fig 4:**
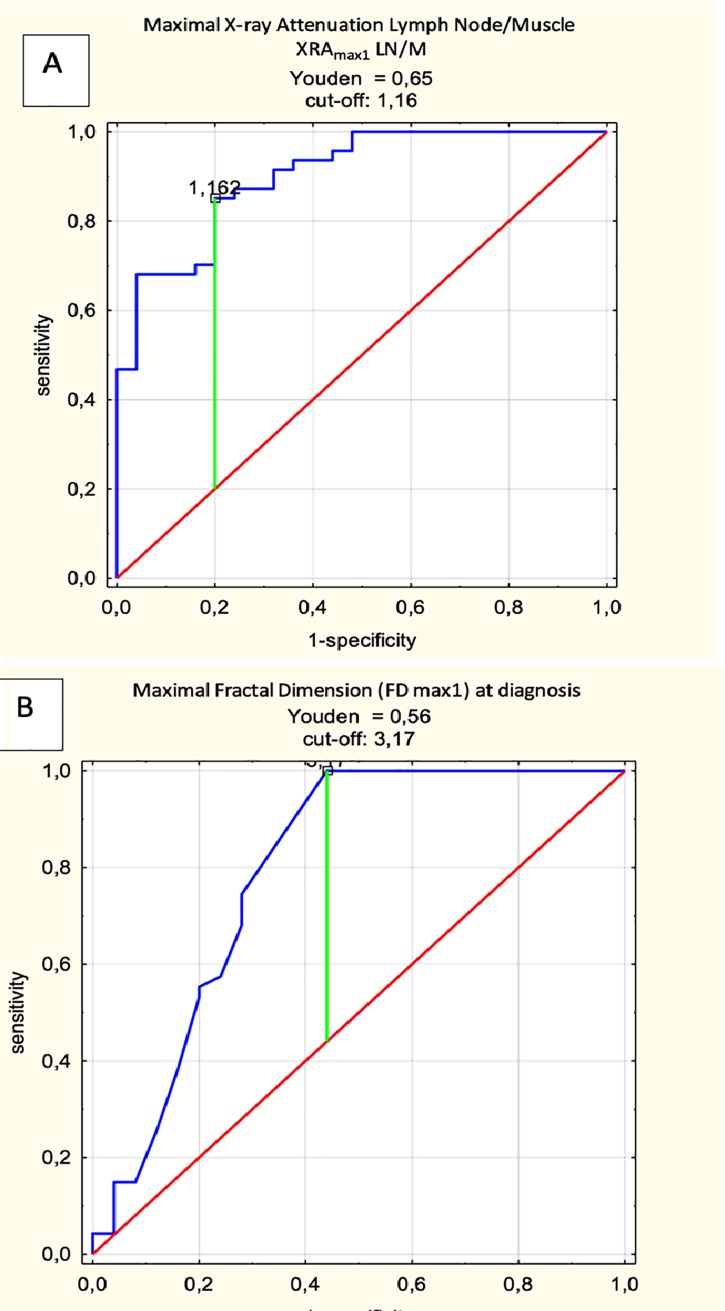
Receiver operating characteristic (ROC). Initial 18F-FDG-PET/CT, PET(-) vs PET(+) in the training set (n = 71). Maximal X-ray Attenuation Lymph Node/Muscle (A) and Maximal Fractal Dimension (B) at diagnosis in the training set (n = 71).

In the next step, the cut-off values of XRA_max_ LN/M and FD_max_ were determined using receiver operating characteristic analysis (ROC) implementing the Youden index, which can differentiate PET(+) and PET() nodes with the greatest accuracy. The following cut-off values were obtained: XRA_max_ LN/M = 1.16 (AUC 0.92; 95% AUC 0.83–0.97; sensitivity 0.85; specificity 0.85; accuracy 0.83) and FD_max_ = 3.17 (AUC 0.79; 95% AUC 0.67–0.92; sensitivity 1.0; specificity 0.56; accuracy 0.85) (Fig 4).

Based on obtained cut-off values for XRA_max_ LN/M and FD_max_, a point scale was created, in which each value of XRA_max_ LN/M of >1.16 and each value of FD_max_ of >3.17 was given 1 score. The results for the training set are shown in Table 3.

**Table 3:** Results of the XRA_max_ LN/M + FD_max_ test in the training and test sets.

training set	2pt	1pt	0pt	total	test set	2pt	1pt	0pt	total
**PET(-)** (no LN)	4	8	13	25	**PET(-)** (no LN)	1	9	30	40
**PET(+)** (no LN)	40	7	0	47	**PET(+)**(no LN)	23	8	0	31
**total**	44	15	13	72	**total**	24	17	30	71

LN, lymph node.

Based on this scale, nodes with a score of 2 points were determined as potentially PET(+), nodes with a score of 0 points were determined as PET(-), and nodes with a score of 1 point as undefined. The results of the test are presented in Table 4. The undefined nodes (1 point) with the unidentifiable status of PET constituted 21% of all nodes analyzed. Overall in the training set, the compatibility of the results of the XRA_max_ LN/M + FD_max_ test (HUFRA–Hounsfield Units FRActal dimension) with 18F-FDG-PET/CT examination was 74% of the nodes analyzed whereas after excluding nodes with a score of 1 point, it was 93%.

**Table 4:** Parameters of the HUFRA test (XRA_max_ LN/M + FD_max_) in the training and test sets.

	sensitivity	specificity	PPV	NPV	accuracy
training set.all LN (n = 72)	0.85	0.52	0.68	0.46	0.74
training set without LN with 1 points (n = 57)	1.0	0.76	0.91	1.0	0.93
test set. all LN (n = 71)	0.74	0.75	0.56	0.64	0.75
test set without LN with 1 points (n = 54)	1.0	0.97	0.96	1.0	0.98
training and test sets all LN (n = 143)	0.81	0.66	0.63	0.57	0.74
training and test sets without LN with 1 points (n = 111)	1.0	0.90	0.93	1.0	0.95

LN, lymph node; PPV, positive predictive value; NPV, negative predictive value.

During the follow-up step, the HUFRA specification mentioned above was applying to 71 nodes from the tested group. The accuracy of this test was rated as 75%. After including nodes with a score of 2 and 0 points, the accuracy reached 98%. In the observed group, the percentage of nodes with unidentifiable status was 24%. The results and test parameters are presented in Tables 3 and 4.

In summary, after excluding nodes with unidentifiable status with 1 point score, (22.5% of all nodes analyzed in both groups), the HUFRA test accurately qualified 95% of the remaining nodes with a score of 2 and 0 points as PET(+) or PET(-).

## Discussion

The attempts of using FD for improving interpretation of radiological examinations have been undertaken in oncology already before, e.g., in breast [31], lung [32], and central nervous system cancers [33]. It is confirmed that normal tissues have different FD than cancer tissues. This difference arises from a different arrangement of blood vessels and texture of the whole tumor mass (e.g., areas of hypoxia develop secondary to the pathological vascularization pattern, and have a profound impact on the evolution of the tumor’s stromal microenvironment) [34]. The role and significance of FD in the radiological diagnosis of lymphomas, particularly in CT examinations, remains unclear. In our previous research [11,17], we presented data showing a potential benefit of determination of FD and HU in the native CT study in Hodgkin's lymphoma in children. The results obtained in the present study, using simple measurement during initial CT examination, allow the accuracy of about 75–80% for qualifying lymph nodes as PET(+) or PET(-). Additionally, this assessment can be carried out retrospectively. High concordance between PET and CT data for nodal regions has been determined in pediatric HL and highlighted in previous studies [11]. The method presented in our research cannot be used in the case of lymph nodes with a high value of only 1 of the 2 parameters–XRA_max_ LN/M or FD_max_. In the case of the majority of the remaining nodes with a high or low value of both parameters, sensitivity (100%) and specificity (93%) of the HUFRA test are satisfactory. We are aware that the number of patients enrolled in the current research and the number of lymph nodes analyzed is not significantly high. In order to confirm our findings and to prove that the presented method has universal values, the measurement can be carried out on the potentially larger groups of patients who were under 18F-FDG-PET/CT examinations performed with different devices. Our research data shows the compatibility of the results between the training and test sets. The CT scans results, received by using other devices seem to confirm the statement that evaluated parameters—XRA_max_ LN/M and FD_max_ are independent of parameters of the CT examination.

The 18F-FDG-PET/CT examination has an established role and diagnostic value in HL [35], although some publications report superiority of classical CT examination over 18F-FDG-PET/CT in determining a positive predictive value for disease recurrence (22.9% vs 28.6%) [36]. The HUFRA test we have proposed here is not aimed at replacing this examination since, in HL, 18F-FDG-PET/CT is a gold standard in staging and evaluating the response to CTx. Taking into consideration that the result is solely based on the metabolic imaging (PET) leads to upstaging or downstaging in approximately 15–40% of patients with HL with and impacts management in about 5–15% of them [37,38], HUFRA may be a source of additional unique information [39].

The HUFRA test may also serve as an alternative for patients who did not undergo 18F-FDG-PET/CT examination for various reasons before starting the treatment for HL but had only a standard initial CT. For example, in the situation, when patients are diagnosed with a massive tumor of the mediastinum and clinical manifestations of the superior vena cava syndrome, who will require immediate start of treatment to alleviate life-threatening symptoms. In this group of patients, steroid therapy alone or in combination with CTx may significantly affect the images produced by 18F-FDG-PET/CT during the course of this treatment. Other potential patients who can benefit from the HUFRA test are those who initially were suspected of having the other pathology/diagnosis for which the 18F-FDG-PET/CT examination is not required, but the use of steroids is recommended, e.g. non-Hodgkin's lymphomas. After changing the diagnosis, it is too late to obtain reliable results of 18F-FDG-PET/CT examination. Currently, only few contradictions for 18F-FDG-PET/CT exist. The reliable result can be obtained when the patients are properly prepared for the examination, even in the cases combined with diabetes or other endocrine diseases, affecting carbohydrate metabolism [40]. The HUFRA test may be also useful in patients with unstable blood glucose values, before 18F-FDG-PET/CT examination or in cases suspicious for patient glycemic status, which may influence the result of this examination. Sometimes in the course of HL, it is necessary to perform a thoracotomy in order to sample a lymph node for histology. Previous extensive surgery may alter the result of 18F-FDG-PET/CT examination as well.

The availability of 18F-FDG-PET/CT examination is satisfactory in the developed countries. However, its unit costs of €600–700 and the necessity of using advanced technical infrastructure are a serious barrier to the availability of this examination in poorer countries. If the result presented in the study could be confirmed on a larger group of patients, the HUFRA test along with native CT examination could be used as an alternative method to improve quality of HL staging in countries with limited accessibility to 18F-FDG-PET/CT. Another potential application of the HUFRA test is its use in automatic CT image detection systems, which facilitates and speeds up the interpretation of CT scans.

Due to limited availability of 18F-FDG-PET/CT results with a sufficient number of PET(+) lymph nodes performed to evaluate response to CTx after 2 cycles, the present study did not attempt to assess the HUFRA test in evaluating the response to CTx. The authors are planning such an analysis as the next phase of the research.

Despite the increasing availability of modern imaging techniques, in addition to rise information value are more secure in the pediatric population (e.g., 18F-FDG-PET/MR), CT examination remains the gold standard for the assessment of lungs. For this reason, it is still performed in children and adolescents with HL as part of the initial diagnosis. The HUFRA test, developed by authors and presented here, does not require additional radiation exposure and special technical devices. It is based only on the existing data which is included in routinely performed CT scans. Additional advantages that the presented method applies its simplicity, low costs, and quick detection of lesions.

Undoubtedly, the assessment of the clinical usefulness of the HUFRA test requires further research on larger groups of patients who had been subjected to 18F-FDG-PET/CT examinations in various conditions. However, the results obtained by our team are really promising that, in our opinion, it is worth undertaking further research in this scope.

## Conclusion

The difference in XRA_max_ LN/M ratio (based on determination of the maximal HU value) and FD_max_ of lymph nodes from pediatric patients with HL in the CT component of 18F-FDG-PET/CT examination between PET(+) nodes (4–5 points on the Deauville scale) and PET(-) nodes (1–3 on the Deauville scale) is significant. Based on this property, the HUFRA test was developed. The HUFRA test can identify about 70–80% of analyzed lymph nodes as PET(+) or PET(-) with high accuracy. The HUFRA test, if its potential effectiveness is confirmed in further studies, will be able to apply to selected cases, e.g. in patients who were not subjected to 18F-FDG-PET/CT examination before the treatment or unreliable result of 18F-FDG-PET/CT. This test can also be used in health care facilities with limited access to 18F-FDG-PET/CT examination.

## Supporting information

S1 Appendix Clinical data and SUV_max_, XRA_max_ LN/M, FD_max_ values in patients from training set. ("A") and test set ("B").
(XLSX)
